# Construction and utilization of a script concordance test as an assessment tool for dcem3 (5th year) medical students in rheumatology

**DOI:** 10.1186/1472-6920-13-166

**Published:** 2013-12-13

**Authors:** Sylvain Mathieu, Marion Couderc, Baptiste Glace, Anne Tournadre, Sandrine Malochet-Guinamand, Bruno Pereira, Jean-Jacques Dubost, Martin Soubrier

**Affiliations:** 1 Service de Rhumatologie, Centre hospitalier Jacques Lacarin, Vichy, France; 2 DRCI. CHU G Montpied, F-63003 Clermont-Ferrand, France, Univ Clermont 1, Fac Médecine, Clermont-Ferrand F-63003, France; 3 Rheumatology Department, Gabriel Montpied Teaching Hospital, Place H Dunant, Clermont-Ferrand 63000, France

**Keywords:** Script concordance test, Rheumatology, 5th year medical students

## Abstract

**Background:**

The script concordance test (SCT) is a method for assessing clinical reasoning of medical students by placing them in a context of uncertainty such as they will encounter in their future daily practice. Script concordance testing is going to be included as part of the computer-based national ranking examination (iNRE).

This study was designed to create a script concordance test in rheumatology and use it for DCEM3 (fifth year) medical students administered via the online platform of the Clermont-Ferrand medical school.

**Methods:**

Our SCT for rheumatology teaching was constructed by a panel of 19 experts in rheumatology (6 hospital-based and 13 community-based). One hundred seventy-nine DCEM3 (fifth year) medical students were invited to take the test. Scores were computed using the scoring key available on the University of Montreal website. Reliability of the test was estimated by the Cronbach alpha coefficient for internal consistency.

**Results:**

The test comprised 60 questions. Among the 26 students who took the test (26/179: 14.5%), 15 completed it in its entirety. The reference panel of rheumatologists obtained a mean score of 76.6 and the 15 students had a mean score of 61.5 (p = 0.001). The Cronbach alpha value was 0.82.

**Conclusions:**

An online SCT can be used as an assessment tool for medical students in rheumatology. This study also highlights the active participation of community-based rheumatologists, who accounted for the majority of the 19 experts in the reference panel.

A script concordance test in rheumatology for 5^th^ year medical students

## Background

The French national ranking examination (NRE) at the end of the second cycle of medical studies is in the process of reform [1]. Indeed, this examination is no longer considered relevant because students’ scores may differ by a matter of a few points (7800 students within 400 points in 2011) or a few dozen points, which leads to a selection based on criteria such as year of birth or score on the first dossier. As medical school enrolment is continually on the rise, this problem will be encountered ever more frequently. Furthermore, the NRE cannot adequately assess the quality of the students’ future clinical competence because, in particular, it does not confront them with the diagnostic uncertainty that is found in the real-life setting [1].

Starting in 2016, the new version of the NRE will be administered to medical students, i.e. those who are currently in their third year of the general medical sciences training diploma (DFGSM3) [1]. This new examination, called the computer-based NRE, will comprise more numerous and more varied tasks in order to confer increased accuracy to the final ranking of the 8–10,000 students who take the examination. The NRE will thus be put to the service of medical training.

The tasks in this new iNRE will include progressive clinical cases, simple open and closed questions, critical review of an article, and script concordance tests (SCT).

The script concordance test (SCT) is a method for assessing clinical reasoning of medical students in the context of uncertainty that characterizes their future daily practice [2-6]. The students are confronted with a clinical situation that points toward a diagnosis, a treatment or an order for further investigations. They are then given new information and asked whether it will support, modify or have no effect on the proposed hypothesis, according to a 5-anchor Likert scale. Their responses are compared with those of a panel of experts who also completed the test. The creation and development of an SCT must involve the recruitment of a sufficiently large panel of experts — at least fifteen — to ensure representativeness. In addition, Petrucci et al. recently emphasized the importance of using specialty-specific experts for bolstering the validity of interpretations of SCT scores. They showed that the examinees’ scores increased progressively with level of training, with the use of specialty-specific panellists [7].

A computer, internet connection and online platform suffice to make an SCT test feasible, as shown in the study by Kania et al. [8]. Van Bruggen et al. recently confirmed that SCTs were suitable for computer-based assessment [9].

The objective of this study was to create such a test in the rheumatology department of the Clermont-Ferrand medical school and to administer it online via an internet platform. Here we describe the different steps we used to construct the clinical scenarios and propose the test to a medical student cohort.

## Methods

### Ethics statement

The present research is not considered as biomedical research according to the French legislation: Law n°2004-806 (09/08/2004) related to politics in public health (L1121-1). Published in: *Journal Officiel de la République Française*, 11/08/2004. Available http://www.legifrance.gouv.fr/affichCodeArticle.do?idArticle=LEGIARTI000025104470&cidTexte=LEGITEXT000006072665&dateTexte=20121126&fastPos=5&fastReqId=5261883&oldAction=rechCodeArticle. Accessed 2 April 2013. Therefore, no ethical approval was required and all data has been analysed anonymously.

### Creation of vignettes

Our test was created in accordance with the guidelines for construction of SCTs [10-13]. Each scenario corresponds to a simple clinical situation commonly encountered in daily practice but which poses a problem because it lacks the information needed to establish a diagnosis or propose a diagnostic or treatment option with certainty. SM wrote each vignette and question but did not participate as a panel expert to avoid bias.

The different domains covering the discipline of rheumatology were addressed through 87 questions: mechanical disorders (osteoarthritis and disc diseases), rheumatic diseases (rheumatoid arthritis, spondylarthropathies), microcrystalline diseases (gout, chondrocalcinosis), inflammatory disorders (polymyalgia rheumatica, Sjogren’s syndrome), infectious diseases (septic arthritis), and osteoporosis. Table 1 summarizes the number of vignettes per rheumatologic domain and the number of questions per vignette. In each diagnostic question, a diagnostic hypothesis is shown in the first column. The second column presents new clinical or laboratory findings that might modify the initial hypothesis (Table 2). Students also had to think about questions of treatment (Table 2).

**Table 1:** Number of vignettes per rheumatologic domain and number of questions per vignette

**Rheumatologic domain**	**Number of vignettes**	**Number of questions**
Rheumatic diseases (rheumatoid arthritis, spondylarthropathies)	6	21
Mechanical disorders (osteoarthritis and disc diseases)	4	12
Inflammatory disorders (polymyalgia rheumatica, Sjogren’s syndrome)	2	10
Microcrystalline diseases (gout, chondrocalcinosis)	2	8
Infectious diseases (septic arthritis)	2	6
Osteoporosis	2	3

**Table 2:** Examples of vignettes with diagnostic and treatment questions used in the script concordance test in rheumatology

• Mrs. Pervenche, 52 years old, consults for pain and inflammation in both wrists and in the two second MCPs for the past 2 months. You find synovitis in these four joints.		
If you were thinking of…	And then if you were to find…	This hypothesis would become:
Making a diagnosis of rheumatoid arthritis	Negative for rheumatoid factor and anti-CCP antibodies	
Making a diagnosis of rheumatoid arthritis	A line of chondrocalcinosis on radiographs of the hand at the level of the triangular carpal ligament	
• Mr Xavier, 68 years old, comes in for his first viscosupplementation injection in the left knee. He is on oral anticoagulants for atrial fibrillation.		
If you were thinking of…	And then if you were to find…	This hypothesis would become:
Administering the injection	Previous day’s INR of 2.9	

Scenarios that the experts judged unclear (n = 11) or which did not contain any uncertainty, i.e. all panel members gave the same answers (n = 6), were discarded.

The SCT contained a total of 18 vignettes, including 47 diagnostic questions and 13 treatment questions. There were no investigational questions, i.e. options for additional investigations. We tailored the construction of our SCT to the level of the examinees (fifth year students), and therefore did not include investigation-type questions.

### Construction of the reference panel

In order to achieve stable scores independent of the composition of the panel, it is recommended that 15 to 20 experts be recruited [14]. All the experts included in this study are specialists in rheumatology. Six physicians from the Clermont-Ferrand rheumatology department (4 hospital practitioners and 2 senior registrars) completed the test. Among the 24 invitations to community-based rheumatologists from the Auvergne region, 13 (54%) agreed to take the test. Thus, a total of 19 experts answered the 60 questions.

### Students

Rheumatology is taught during the fifth year of medical studies (DCEM3). The 179 DCEM3 students were therefore invited to participate in this SCT after having taken the rheumatology examination, in order to ensure that they had all taken the rheumatology module. These students are not concerned by the upcoming NRE reform which includes script concordance testing. They were given information about the advantages of the SCT as an assessment tool for their future professional activity. This SCT was not a certifying exam in rheumatology and was therefore not mandatory. Students who volunteered to take it could also fill out an assessment questionnaire after taking the test (Additional file 1: Appendix 1).

### Creation and administration of the SCT

To facilitate distribution of the test and receipt of the responses, it was put online on the medical school’s digital platform. Online SCTs for medical students have previously been described in the literature [15,16]. The 179 DCEM3 students received an email invitation to login to their session and take the test online. Two additional email reminders were sent two weeks apart to students who had not yet participated.

### Scoring

The scoring system was based on the principle that any response given by an expert has an intrinsic value, even if it does not agree with the responses of the other experts [17-19]. The experts’ responses were used to attribute a score to each question. For each question, the number of points awarded to the examinees, for each possible response, depended on the number of experts who gave the same response.

For example, in our panel, if 11 of the 19 experts selected the response “-2”, 5 selected “-1” and 3 selected “0”, the value of a student’s response “-2” would be 11/11 = 1 point, that of response “-1” would be 5/11 = 0.45 points, that of “0” would be 3/11 = 0.27 points, and that of responses “+1” and “+2” would be 0 points.

The global SCT score was obtained by adding the scores for each question, and then transformed into a 100-point scale [20]. For calculation purposes, we used the software available on the website of the University of Montreal (http://www.cpass.umontreal.ca/tcs.html).

### Statistics

Mean scores for each students and experts were expressed as the mean ± standard deviation; these scores were compared with a Mann-Whitney test for non-normally distributed data. To evaluate differences between groups, *p* < 0.05 was considered statistically significant.

Test reliability was estimated by the Cronbach alpha coefficient. This statistical index (values between 0 and 1) indicates greater homogeneity as values approach 1. When evaluating methods, good reliability is indicated when the coefficient is ≥ 0.80. Test quality is also estimated by the number of ‘bad’, ‘fair’ and ‘good’ items. The calculator computes an item analysis for each question regarding its ability to discriminate between students and its impact on overall reliability. A bad item is defined by a correlation coefficient < 0.10, a ‘fair’ item by a coefficient between 0.10 and 0.20, and a ‘good’ item by a coefficient > 0.20 [21].

## Results

Twenty-six students took the SCT and sent in their responses. This corresponds to a response rate of 14.5% out of a total of 179 students. However, close to 40% of these students (n = 72) never log on to the online platform in rheumatology, whether it be to see the SCT questions or to consult their coursework or clinical cases.

Fifteen of the 26 students completed the test by answering all 60 questions. Nine students answered fewer than 10 questions and two answered 32 and 46 questions, respectively.

**Figure 1:**
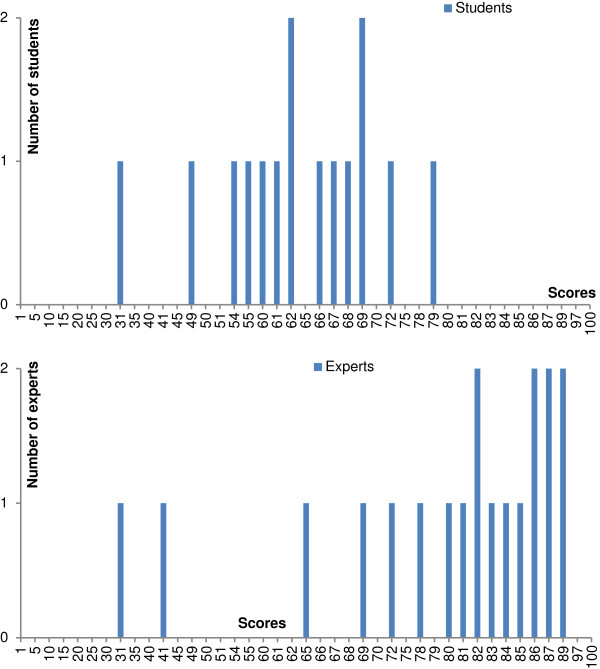
**Distribution of scores for the two groups: students (n=15) and experts (n=19).** The maximum score is equal to 100.

The analysis concerned only the 15 students who completed the entire test. The mean score was 76.6 for the expert panel and 61.5 for the students (p = 0.001) (Table 3 and Figure 1). The complete SCT had a Cronbach alpha coefficient of 0.82 and comprised 11 bad items, 6 fair items and 43 good items.

**Table 3:** Scores of experts and students on the script concordance test

**Scores**	**Expert rheumatologists (n = 19)**	**Students (n = 15)**
Mean	76.6	61.5
Standard deviation	15.7	11.4
Minimum	31.3	30.9
Maximum	89.1	79.0
Confidence interval (95%)	73.0–80.2	58.6–64.5

Seven of the 26 students completed the test assessment questionnaire. Five (71%) did not know about the existence of script concordance tests but six (86%) recognized its instructional value. All the students felt uneasy and unfamiliar with the format of the questions but all agreed that they would participate in another SCT.

## Discussion

Twenty-six DCEM3 (5th year) medical students participated in the SCT in rheumatology on the online platform of the Clermont-Ferrand medical school, demonstrating that online test administration is possible. This SCT has good reliability, with a Cronbach alpha coefficient greater than 0.80, and good construct validity with a higher mean score in experts than in students.

The SCT is generally used for training and evaluation during the third cycle of medical studies. The test has been used by interns in general medicine and other specialties [22-25]. Caire et al. have used it as a training tool for neurosurgery interns since 2003 and conclude that it is an interesting and easily accessible means of self-assessment that merits more widespread use [26]. Lubarsky et al. demonstrated the validity of their SCT in neurology: neurology residents scored higher than medical students but lower than neurologists [27], as also reported by Sibert et al. in the field of urology [28]. The use of script concordance testing in second cycle medical students has already been described in the literature and the method has shown promise as an assessment tool, as in our study [29-36]. For example, Caire et al. administered a 30-item SCT in neurosurgery to 75 fourth-to-sixth year medical students and found an association between the mean scores and the level of training: 51.8 for fourth year, 54.9 for fifth year and 59.8 for sixth year medical students. These students all received the same theoretical instruction in neurosurgery, indicating that this test also assesses the experience acquired during clinical training [37]. Similarly, Humbert et al. found that their SCT successfully differentiated between second and fourth year medical students since the former had a lower score [38]. Nonetheless, it is important to adapt the vignettes and questions to the level of instruction of the students, otherwise the test quickly becomes useless or even counterproductive. Duggan et al. administered a script concordance test comprising 51 cases with 158 questions across seven different medical specialties as a summative assessment of students. Twenty-three sixth year students and 132 fifth year students took the test and only four failed, i.e. their score was not within four standard deviations of the expert mean score [39].

A noteworthy finding in our study concerns the high participation rate of community-based rheumatologists, over half of whom agreed to participate despite their busy schedules. This bears witness to their motivation and suggests that clinical rotations, which are increasingly numerous in hospitals, could also be carried out in community medicine, as in the training module for general medicine interns. One of the limitations of the SCT is to recruit a large enough panel of experts. A panel consisting of 15 experts might be considered too large for hospital departments, where the number of physicians is usually lower; this could constitute a hindrance to the administration of SCTs. Our study provided a solution by inviting community-based rheumatologists: the insufficient number of six hospital physicians was easily completed by 13 community-based physicians. The heterogeneity of the panel is not a problem. On the contrary, as shown by Pleguezuelos et al., the use of a composite panel even including international experts still yields high psychometric quality [40]. In our study, two panel members had surprising scores (31 and 41), which are clearly lower than those of other experts. If we exclude these two lowest scores, we find a mean score of 80.7 ± 6.9 in the panel of 17 experts. These new statistics are more consistent with the literature. The two lowest scores lower the panel’s mean, boost SD and underestimate the good construct validity of our SCT. However, experts with deviant responses should not be excluded, since the resulting measurement error is negligible if the panel size is sufficient (> 15) [41]. The mean scores of the 19-member panel and the 17-member panel are very close (76.6 vs. 80.7).

There are limitations to this study. The principal limitation is related to the small cohort of participants. Only 15% (26/179) of the invited students took the test and 42% (11/26) did not complete it. This precludes a generalization of our findings to the group of students as a whole. Nonetheless, our main objective — to create and administer an online SCT — was achieved. The low participation rate may be explained by two factors. First, the test was proposed to students currently in their fifth year and who, a priori, will not be concerned by the computer-based NRE reform of 2016. The fact that they will not have a SCT in their own NRE might explain their lack of motivation to participate in this optional test. Furthermore, according to the statistics of the online platform in rheumatology, it was found that close to 40% of students never visit the site to consult their courses, clinical cases or other documents. This reflects literature data showing that the response rates to internet-based surveys are highly variable, ranging from excellent (94%) to much lower than ours (9%) [42,43]. Secondly, 42% of our participants did not complete the entire test. Nine of the 26 participants completed fewer than 10 out of 60 questions and four did not answer any questions. It is possible that these students logged in out of curiosity without the intention to complete the test. However, we cannot rule out that they were discouraged by the question format which they had never encountered before. Moreover, all the students who returned the test assessment questionnaire noted that they were unfamiliar or uncomfortable with the question format. Therefore it is very important to establish the rules for SCTs and to provide clear instructions and information to the students, who are not acquainted with this type of learning.

## Conclusion

To our knowledge, this is the first study of the creation and administration of a script concordance test to assess clinical reasoning in the field of rheumatology among fifth year medical students. A total of 26 students took the test on the online platform of the Clermont-Ferrand medical school and 15 completed it. Despite the low participation rate, the possibility of using this internet-based SCT was demonstrated. One important issue, and perhaps a limitation of creating such a test, is the cost and time needed to develop the questions and recruit panel members. We think that online administration is the most difficult part in the development of an SCT. In our case, the internet platform of the Faculty of Medicine already existed, which made it possible to distribute the test to those students who use it. Creating the vignettes and questions takes time but can be done by one person, as in our study. The University of Montreal software is very useful for scoring the test. If the instructions are followed, it is very easy to use.

## Competing interests

The authors declare that they have no competing interests.

## Authors’ contributions

All authors contributed to devising the study, interpreting the data, and writing the paper. SM collected and analysed the data and wrote the first draft of the paper and BP performed the statistical analysis. All authors approved the final version.

## Pre-publication history

The pre-publication history for this paper can be accessed here:

http://www.biomedcentral.com/1472-6920/13/166/prepub

## Supplementary Material

Additional file 1: Appendix 1 Students’ assessment questionnaire about the script concordance test. Click here for file
