# Supplementary material for: Construction and utilization of a script concordance test as an assessment tool for dcem3 (5th year) medical students in rheumatology
Source: BMC Med Educ. 2013 Dec 13;13:166. doi: 10.1186/1472-6920-13-166 (PMC3878954; doi:10.1186/1472-6920-13-166)
Supplement: Additional file 1: Appendix 1 — Students’ assessment questionnaire about the script concordance test. [file 1472-6920-13-166-S1.doc]

Additional file 1: Appendix 1. Students’ assessment questionnaire about the script concordance test

1. Did you know about the existence of these script concordance tests?

□ No □ Yes

1. Are you satisfied to have participated in this test?

□ No, not at all □ No, not really □ Yes, somewhat □ Yes, completely

1. Did you understand the instructional value of taking such a test?

□ No, not at all □ No, not really □ Yes, somewhat □ Yes, completely

1. Did you feel at ease when completing this test?

□ No, not at all □ No, not really □ Yes, somewhat □ Yes, completely

1. Were you uncomfortable with the format of the questions?

□ No, not at all □ No, not really □ Yes, somewhat □ Yes, completely

1. Do you think this type of test is useful for your future medical practice?

□ No, not at all □ No, not really □ Yes, somewhat □ Yes, completely

1. Do you think this type of test should be part of the NRE?

□ No, not at all □ No, not really □ Yes, somewhat □ Yes, completely

1. If you were invited to participate in another script concordance test, would you accept?

□ No, not at all □ No, not really □ Yes, somewhat □ Yes, completely

1. Would you recommend this test to your colleagues who have not yet taken it?

□ No □ Yes
